# Effect of Electrical Stimulation Conditions on Neural Stem Cells Differentiation on Cross-Linked PEDOT:PSS Films

**DOI:** 10.3389/fbioe.2021.591838

**Published:** 2021-02-12

**Authors:** Laura Sordini, Fábio F. F. Garrudo, Carlos A. V. Rodrigues, Robert J. Linhardt, Joaquim M. S. Cabral, Frederico Castelo Ferreira, Jorge Morgado

**Affiliations:** ^1^Department of Bioengineering and iBB – Institute for Bioengineering and Biosciences, Instituto Superior Técnico, Universidade de Lisboa, Lisbon, Portugal; ^2^Department of Bioengineering and Instituto de Telecomunicações, Instituto Superior Técnico, Universidade de Lisboa, Lisbon, Portugal; ^3^Department of Chemistry and Chemical Biology, Center for Biotechnology and Interdisciplinary Studies, Rensselaer Polytechnic Institute, Troy, NY, United States

**Keywords:** electrical stimulation, neural stem cells, neuronal differentiation, ReNcell VM, conjugate polymer, electroconductive material, PEDOT:PSS, cross-linking

## Abstract

The ability to culture and differentiate neural stem cells (NSCs) to generate functional neural populations is attracting increasing attention due to its potential to enable cell-therapies to treat neurodegenerative diseases. Recent studies have shown that electrical stimulation improves neuronal differentiation of stem cells populations, highlighting the importance of the development of electroconductive biocompatible materials for NSC culture and differentiation for tissue engineering and regenerative medicine. Here, we report the use of the conjugated polymer poly(3,4-ethylenedioxythiophene) doped with polystyrene sulfonate (PEDOT:PSS CLEVIOS P AI 4083) for the manufacture of conductive substrates. Two different protocols, using different cross-linkers (3-glycidyloxypropyl)trimethoxysilane (GOPS) and divinyl sulfone (DVS) were tested to enhance their stability in aqueous environments. Both cross-linking treatments influence PEDOT:PSS properties, namely conductivity and contact angle. However, only GOPS-cross-linked films demonstrated to maintain conductivity and thickness during their incubation in water for 15 days. GOPS-cross-linked films were used to culture ReNcell-VM under different electrical stimulation conditions (AC, DC, and pulsed DC electrical fields). The polymeric substrate exhibits adequate physicochemical properties to promote cell adhesion and growth, as assessed by Alamar Blue® assay, both with and without the application of electric fields. NSCs differentiation was studied by immunofluorescence and quantitative real-time polymerase chain reaction. This study demonstrates that the pulsed DC stimulation (1 V/cm for 12 days), is the most efficient at enhancing the differentiation of NSCs into neurons.

## Introduction

The World Health Organization (WHO) predicted that by 2040 neurodegenerative diseases will be the second cause of death worldwide after cardiovascular diseases (Gammon, 2014). The steady increase in the number of diagnosed cases of neurodegenerative diseases accompanies the increase in life expectation. In the USA alone, between 2000 and 2017, the number of declared deaths attributed to Alzheimer's disease increased by 145% (8.5% a year) (Alzheimer's Association, 2019). A common pattern to all neurodegenerative diseases is a genetic, cellular, and/or neural circuit dysregulation that leads to progressive and yet massive neuronal death (Heemels, 2016). The conventional pharmacological and non-pharmacological approaches available are only palliative and cannot halt or reverse the disease. It is therefore pivotal to develop more effective treatments and/or new therapeutics able to tackle neurodegenerative diseases (Heemels, 2016; Erkkinen et al., 2018).

Tissue engineering strategies are promising for the definitive cure of neurodegenerative diseases through tissue regeneration. Orchestrating such strategies is no trivial task, as the complexity and the widespread of brain areas affected by these diseases calls for a multidisciplinary approach. As such cell-based approaches, including encapsulated cell technology, drugs and growth factor delivery, genetic manipulation, and also the use of bioactive materials must be integrated for success (Borlongan et al., 1999; Tresco, 2000).

Despite the difficulties in isolating, neural stem cells (NSCs) are attractive for cell-based therapies because of their potential ease of propagation and manipulation, their ability to migrate to disease affected sites and their capacity of differentiating into any neural cell type required (Harrower and Barker, 2004). However, the difficulty to isolate these cells from tissues has driven several researchers to study the development of effective protocols to differentiate induced pluripotent stem cells (iPSCs) into NSC (Galiakberova and Dashinimaev, 2020, Fernandes et al., 2015). NSC *in vitro* culture and survival under transplantation can be enhanced by the use of appropriate scaffolds, which can replicate the cues of the NSC niche and promote differentiation toward specific lineages. Some of these cues include topography (Qi et al., 2013), mechanical (Pathak et al., 2014), and electrical stimuli (Zhu et al., 2019).

The *in vivo* electrical stimulation of specific brain areas stands at the cutting edge of potential therapeutic approaches. For example, deep brain stimulation (DBS) is a powerful clinical therapeutic technique that can alleviate movement disorders in patients that no longer respond satisfactorily to pharmacological management (Kühn and Volkmann, 2017). It requires the implantation of an array of electrodes in specific areas of the brain for the delivery of electrical pulses. These can be used to block abnormal neural activity associated with Parkinson's disease and help to normalize cell homeostasis (Benabid, 2003).

Electrical stimulation can also be harnessed to enhance the neural differentiation of NSCs cultured *in vitro* (Ghasemi-Mobarakeh et al., 2009; Pires et al., 2015; Yang et al., 2017). Electroconductive scaffolds with good biocompatibility profile were needed to achieve such goals. Among the electroconductive materials available to produce such platforms, the most used are metals and organic materials, namely graphene, and conjugated polymers. Conjugated polymers present numerous advantages toward neural tissue engineering applications, namely: (1) they can be processed into any desired 3D-shape (Garrudo et al., 2019a,b; Kayser and Lipomi, 2019); (2) can be easily functionalized allowing the tailoring of their mechanical, chemical, and electrical properties; (3) can exhibit high electroconductivity values, approaching those of metals; and (4) combine ionic and electronic conductivity, improving the “quality” of the interfaces with biological tissues (Rivnay et al., 2014; Inal et al., 2018; Goel et al., 2019; Pace et al., 2019). While electronic conductivity promotes higher current flow across the material, ion conductivity may prove to be essential to interface with tissues, as electrical signals in the human body are predominantly associated to ion currents. Therefore, the use of the mixed ionic-electronic of these doped conjugated polymers systems, rather than metals, allows the creation of an interface with an ion “transduction” between external electronic devices/systems and the biological tissues, where electrical signaling occurs via ionic conduction. As an example, the work of Inácio et al. (2020) evidences improved electrical interfacing of neural probes with PEDOT:PSS, which allows the recording of cellular electrical signals with higher signal-to-noise ratio than metals.

The most studied conjugated polymers for neural tissue engineering applications include poly(pyrrole) (PPy), poly(aniline) (PANI), and poly(3,4-ethylenedioxythiophene):polystyrene sulfonate (PEDOT:PSS) (Du et al., 2020). All of these have been proven to be biocompatible for neural applications, with minimal inflammatory response (Guarino et al., 2016; Garrudo et al., 2019a). Upon charge-transfer doping, their electroconductivity can reach values up to 7.5 × 10^3^ S cm^−1^ (Kaur et al., 2015). PEDOT:PSS offers significant advantages when compared to PPy and PANI, including superior thermal and electrochemical stability, charge capacity, low impedance at the interface with electrolytes and combined electronic and ionic conductivity (Collazos-Castro et al., 2010). This justifies the use of PEDOT:PSS in the development of coatings for electrodes capable of being used in deep brain stimulation (Balint et al., 2014). For example, coating metal electrodes with PEDOT:PSS can enhance the electrode's performance by decreasing the interfacial impedance and increasing the charge storage capacity (Bodart et al., 2019). The *in vivo* performance of neural signal recording electrodes coated with PEDOT and carbon nanotubes was tested and a consistent reading signal was found after 11 days of implantation (Alba et al., 2015). Moreover, PEDOT:PSS can also be employed in the design of electroactive scaffolds (Wang et al., 2017; Tomaskovic-Crook et al., 2019).

Cross-linking is the simplest approach to improve the structural stability of PEDOT:PSS-based substrates to support cell culture. Two main cross-linking agents have been reported: (3-glycidyloxypropyl)trimethoxysilane (GOPS) and divinylsulfone (DVS). Neural cells differentiated on PEDOT:PSS films cross-linked with GOPS were found to elongate and exhibit longer neurites after electrical stimulation by pulsed direct current (pulsed DC) (Pires et al., 2015). Mantione et al. (2017) have demonstrated that the use of DVS as PEDOT:PSS cross-linker shows full biocompatibility and better support for neuro-regeneration when compared to GOPS cross-linked material.

The effects of electrical stimulation on NSCs have been widely investigated (Yamada et al., 2007; Chang et al., 2011; Huang et al., 2015; Du et al., 2018; Zhu et al., 2019). Several studies have reported that the application of an extracellular DC field can direct the migration of NSCs and promote neurite outgrowth cathodically (Feng et al., 2012; Meng et al., 2012; Li et al., 2014; Zhao et al., 2015; Hayashi et al., 2016; Yao and Li, 2016). Exposure of NSCs to 0.53 or 1.83 V m^−1^ is associated with increased cell elongation, longer neurites, mature neuronal morphology and increased β*III-Tubulin* expression (Kobelt et al., 2014). Additionally, cells populations derived from stimulated NSCs showed an increase in intracellular Ca^2+^ concentration during stimulation, a signal for the presence of functional neurons. Pulsed electrical stimulation (0.25 mA cm^−2^, biphasic waveform of 100 μs pulses) through laminin-coated PPy electroconductive films can promote the differentiation of NSCs to neurons (Stewart et al., 2015). Moreover, the neurons obtained exhibited clustering and increased neurite growth (longer neurites and greater branching). The application of an alternate current (AC) was shown to induce a morphological change of PC12 cells, promoting neurite growth even in the absence of nerve growth factor (NGF) (Kimura et al., 1998).

Although the reported literature shows the numerous benefits of electrical stimulation on promoting NSC differentiation into neurons, an optimized protocol has not yet been established. Therefore, the main aim of this study is to investigate the effects of applying different types of current flow (AC, DC, or pulsed DC) on NSCs growth and differentiation, when cultivated on electroconductive PEDOT:PSS films. To do this, PEDOT:PSS platforms were optimized and the effect of the cross-linkers GOPS and DVS on polymer properties was compared.

## Materials and Methods

### Cross-Linked PEDOT:PSS Films

Cross-linked PEDOT:PSS films were prepared as follows: glass coverslips were cleaned with acetone and isopropanol under ultrasounds, followed by drying with a nitrogen stream. The surface was then treated with oxygen plasma (PlasmaPrep2, GaLa Instrument), to remove organic residues and increase the hydrophilicity of the surface. PEDOT:PSS dispersion (Heraeus, CLEVIOS P AI 4083, PEDOT:PSS weight ratio 1:6, solids content 1.3–1.7%) was filtered with a 0.45 μm filter before use. Two different solutions were prepared and used to make thin films by spin coating (Spin-Coater KW-4A, Chemat Technology) according to the following protocols:

(i) PEDOT:PSS + GOPS: This dispersion was prepared by adding the dopants ethylene glycol (EG) (added in a 1:4 volume parts, Sigma-Aldrich) and dodecylbenzenesulfonic acid (DBSA) (0.5 μL mL^−1^, Sigma-Aldrich), and GOPS (10 μL.mL^−1^, Sigma-Aldrich) to improve film formation and stability (Pires et al., 2015). The obtained aqueous dispersion was spun-coated at a spinning speed of 1,800 rpm for 60 s. Afterwards, the obtained film samples (PEDOT:GOPS) were annealed at 150°C for 2 min in air.

(ii) PEDOT:PSS + DVS: This dispersion was prepared by adding the dopants EG (1:4 volume parts, Sigma-Aldrich) and DBSA (1 μL mL^−1^, Sigma-Aldrich), and DVS (30 μL mL^−1^, Sigma-Aldrich) to improve film formation and stability (Mantione et al., 2017). The aqueous dispersion was spin coated onto cleaned glass coverslips at a spinning speed of 1,000 rpm for 40 s. Afterwards, the obtained film samples (PEDOT:DVS) were annealed at 50°C for 1 h in air.

### PEDOT:PSS Film Characterization

#### Thickness and Morphology

Film thickness was measured with a Bruker's Dektak® 3.21 Profilometer: a cut in the film was made with a scalpel until reaching the glass substrate and the height of the cut (film thickness) was measured upon surface scanning perpendicularly to the cut. The morphology of the cross-linked PEDOT:PSS films was evaluated using scanning electron microscopy (SEM Hitachi S-2400, Hitachi) at 15 kV, after coating with a thin layer of gold/palladium. Elemental analysis was carried using an EDS Bruker SDD light elements detector.

#### Contact Angle Measurements

Contact angle measurements were performed through the sessile drop method using a Kruss DSA25B goniometer. A drop of distilled water was deposited on the surface of the various PEDOT cross-linked thin films (*n* = 3) and Drop Shape Analysis 4 software was used to take measurements every 5 s for 2 min.

#### Four-Point Probe Electroconductivity Measurement

Four stripes of gold were deposited on the cross-linked PEDOT films by physical vapor deposition (PVD) with an Edwards E306A thermal evaporator, across the entire film and with equal distance from each other. Silver paste (HAZ Electrodrag 1415, Agar) was used to connect the probes to the gold stripes to ease the measurements, performed in triplicate (*n* = 3) and averaged. Upon recording of the potential difference between the two inner contacts for every value of applied current (at the outer contacts), it is possible to derive the slope of the straight line (R = V/I) following Ohm's law. At a constant temperature, the resistance of the sample (R) is proportional to its resistivity (ρ) and to the separation between the two inner contacts (L), and inversely proportional to the cross-section (A) (product of the sample thickness by the sample width), as described by Equation (1):

(1) $$\begin{array}{l} R=\rho \;\times \;\left(L/A\right)\;\left[S^{-1}\;or\;\Omega \right] \end{array}$$

Conversely, it is possible to calculate the conductivity of our sample (σ), as the reciprocal of the ρ value obtained using Equation (2):

(2) $$\begin{array}{l} \sigma =1/\rho \;\left[S\;cm^{-1}\right] \end{array}$$

#### Cross-Linked PEDOT:PSS Films Stability Assay

The assessment of cross-linked PEDOT:PSS stability was performed by immersing the obtained PEDOT:DVS and PEDOT:GOPS films in MilliQ water for 7 and 15 days. At these time points, samples were dried with nitrogen stream and their thickness and conductivity were measured.

### Cell Culture and Characterization

#### NSCs Culture Conditions

ReNcell-VM (Millipore®) is a human neural progenitor cell line derived from the ventral mesencephalon region of the fetal brain and immortalized by retroviral transduction with the v-myc oncogene. ReNcell-VM were first expanded in T-flasks (Falcon®, Corning) previously coated with poly-L-ornithine (PO) (20 μg mL^−1^, Sigma-Aldrich) for 30 min and laminin (LN) (10 μg mL^−1^ in PBS, Sigma-Aldrich) for 4 h at 37°C and 5% CO_2_. After seeding, cells were expanded in N2 medium, consisting of DMEM/F12 with glucose (1.6 g L^−1^, Sigma-Aldrich), N2-supplement (1%, Thermo Fisher), penicillin/streptomycin (1%, Thermo Fisher) and insulin (20 μg mL^−1^, Sigma-Aldrich), and supplemented with EGF (20 ng mL^−1^, Peprotech), FGF-2 (20 ng mL^−1^, Peprotech), and B27 supplement (20 μl mL^−1^, Thermo Fisher).

#### Electrical Stimulation

**Figure 1:**
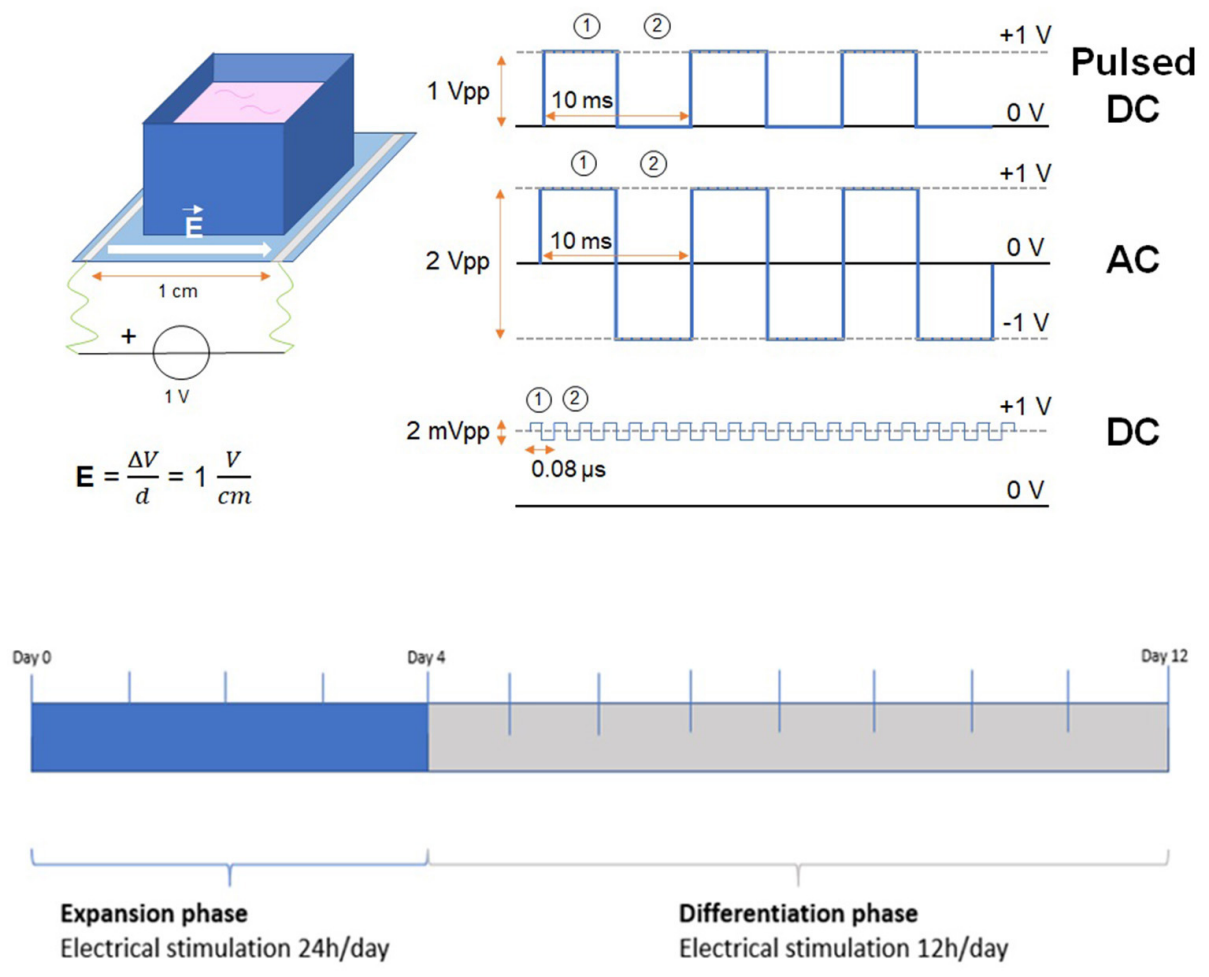
**(Top)** Experimental scheme of the three different types of applied electrical stimulation (pulsed DC, AC, and DC) on cross-linked PEDOT films. **(Bottom)** Timeline of cell expansion and differentiation phases.

Cell culture setups were prepared according to the following procedure. 3D-printed poly(lactic acid) chambers were glued to PEDOT:GOPS films using medical glue (Sylastic A Medical Adhesive, Dow Corning). Copper wires were glued to each end of the PEDOT films with silver paste (HAZ Electrodrag 1415, Agar) and outside the chamber to stimulate the PEDOT films only. An external power supply (Tektronix AFG 1022), connected to the electrodes, was used to apply an electric field across the PEDOT film (Figure 1A). An oscilloscope (Tektronix TBS 200 Digital Oscilloscope) was also connected to the circuit in order to record the applied voltage across the sample.

Setups were sterilized with Anti-anti solution (1%, Thermo Fisher) for 3 h and then coated with poly-L-ornithine and laminin before ReNcell-VM (passage between 20 and 30) seeding (140,000 cells cm^−2^). Cells were let to attach at 37°C and 5% CO_2_ for 1 h before initiating the electrical stimulation. The maximum stimulation threshold value was chosen to avoid the electrolysis of water, which occurs at 1.2 V and can lead to changes in the culture medium pH. The values of frequency and peak duration were set according to the protocols used by Pires et al. (2015). Three different types of electrical stimulation protocols were used in the experiments (Figure 1A):

- *Pulsed DC* voltage of +1 V, with a square wave of 1 V peak-to-peak amplitude (+1 V, 0 V), 100 Hz frequency, 10 ms period;
- *AC voltage* of ± 1 V, applied with a square wave of 2V peak-to-peak amplitude (+1 V, −1 V), 100 Hz frequency, 10 ms period;
- *DC voltage* +1 V, with a square wave of 2 mV peak-to-peak amplitude and an offset of +1 V (+999 mV, +1,001 mV), 12.5 MHz frequency, 0.08 μs period. For the DC voltage, the highest frequency reachable by the power supply and the smallest modulation amplitude of the wave were selected aiming to approximate the output to a DC stimulation.

Cross-linked PEDOT films with no applied electric field and polystyrene cell culture plates (Falcon®, Corning), both coated with poly-L-ornithine and laminin (PO/LN) as previously described, were used as control. Cells were expanded for 4 days under continuous stimulation (24 h), then differentiation was carried on with 12 h continuous stimulation per day for 8 days (Figure 1B).

Differentiation was induced by the withdrawal of growth factors EGF and FGF2 and switching the culture medium to N2B27 to induce neural differentiation. This medium is composed of a 1:1 mixture of N2 medium and B27 medium, this last composed of Neurobasal medium (Thermo Fisher) supplemented with B27 (20 μl mL^−1^, Thermo Fisher), L-glutamine (2 mM, Thermo Fisher), and penicillin streptomycin (0.5%, Thermo Fisher). During the 8 days of the differentiation phase the medium was changed every 2 days.

#### Metabolic Activity Under Electrical Stimulation

Alamar Blue® cell viability reagent was used to study ReNcell-VM metabolic activity under different electrical stimulation conditions during the expansion phase (*n* = 2). Cells were incubated with Alamar Blue® (10% in N2 medium) for 2 h, before sample collection and fluorescence intensity analysis (excitation at 560 nm and emission collected at 590 nm) using Tecan Infinite M200 Pro plate reader.

#### Differentiation Under Electrical Stimulation

After following the different stimulation protocols for the proliferation and differentiation of ReNcells-VM (Figure 1), cell samples were collected for immunocytochemistry and quantitative real-time polymerase chain reaction (qRT-PCR).

##### qRT-PCR

qRT-PCR was performed using SYBR Green® gene expression assays. The genes for β3-tubullin (TUBB3—immature neurons), microtubule-associated protein 2 (MAP2—mature neurons), glial fibrillary acidic protein (GFAP—astrocytes), NESTIN (late NSCs), and (sex determining region Y)-box 2 (SOX2—NSCs) were chosen as the most relevant to be targeted at the end of the differentiation phase (Day 13). Gene expression for each condition was determined using the ΔΔCt method, normalized to the housekeeping gene GAPDH (*n* = 3). The primer sequences can be found in Table 1.

**Table 1:** Primer sequences used for SYBR^®^ Green chemistry-based qPCR.

**Genes**	**Forward primer sequence (5′-3′)**	**Reverse primer sequence (5′-3′)**
GAPDH	GAGTCAACGGATTTGGTCGT	TTGATTTTGGAGGGATCTCG
SOX2	GGGAAATGGGAGGGGTGCAAAAGAGG	TTGCGTGAGTGTGGATGGGATTGGTG
NESTIN	GAAACAGCCATAGAGGGCAAA	TGGTTTTCCAGAGTCTTCAGTGA
TUBB3	CTCAGGGGCCTTTGGACATC	CAGGCAGTCGCAGTTTTCAC
MAP2	GGCATTGAAGAATGGCAGAT	CCCTGTATGGGAATCCATTG
GFAP	CCGCCACTTGCAGGAGTACCAG	TTCTGCTCGGGCCCCTCATGAG

##### Immunocytochemistry

Cell samples were fixed with paraformaldehyde (PFA) 4% for 30 min at room temperature (RT), washed twice with PBS, and then incubated for 30 min at RT with blocking solution, consisting of Normal Goat Serum (10%, Sigma-Aldrich) and Triton-X-100 (0.2%, Thermo Fisher). The primary antibodies used, anti-TUJ1 (mouse, Biolegend) and anti-GFAP (rabbit, Millipore), were first diluted in staining solution, consisting of a 1:2 dilution of blocking solution in PBS, before incubation with the cells at 4°C, overnight. After this, cells were washed three times with PBS and incubated with the secondary antibodies, goat anti-mouse Alexa 488 (Thermo Fisher) and goat anti-rabbit Alexa 546 (Thermo Fisher), for 45 min at RT and protected from light. Finally, cells samples were washed twice with PBS, the nuclei counterstained with DAPI (1 mg mL^−1^, Sigma-Aldrich) for 5 min at RT and again washed twice with PBS. Immunostained cells were visualized using a fluorescence microscope (Leica DMI 3000B equipped with Nikon-AcT1 software). ImageJ software (National Institute of Health) was used to calculate the extent of expression of both differentiation markers. For every analyzed picture, the percentage of area characterized by TUJ1 staining was calculated and divided by the percentage of area characterized by GFAP expression, in order to obtain a quantitative value describing the neuronal/glial differentiation profile in different conditions. At least 7 pictures were analyzed for every condition. The details on the calculations of the ratios, as well as the results of the expression calculated for each biomarker alone, are presented in Supplementary Information 4. Statistical analysis was performed by one-way ANOVA followed by Tukey's HSD Test (*p* < 0.05 for statistical significance).

## Results

### PEDOT:PSS Film Characterization

In this work, both GOPS and DVS cross-linking agents were used to prepare thin conductive PEDOT films, following protocols similar to the ones previously used, for evaluation of long-term stability and characterization of surface properties. Cross-linkers were added to the PEDOT:PSS dispersions before the spin-coating process and annealing of the films was carried to allow the clearance of residual traces of water improving the electrical performance and progression of the cross-linking reactions. The surface of the samples appeared to be flat and homogeneous, as shown in Supplementary Figure 1. The samples were further characterized for surface wettability, thickness, electrical conductivity and long-term water stability (15 days).

**Figure 2:**
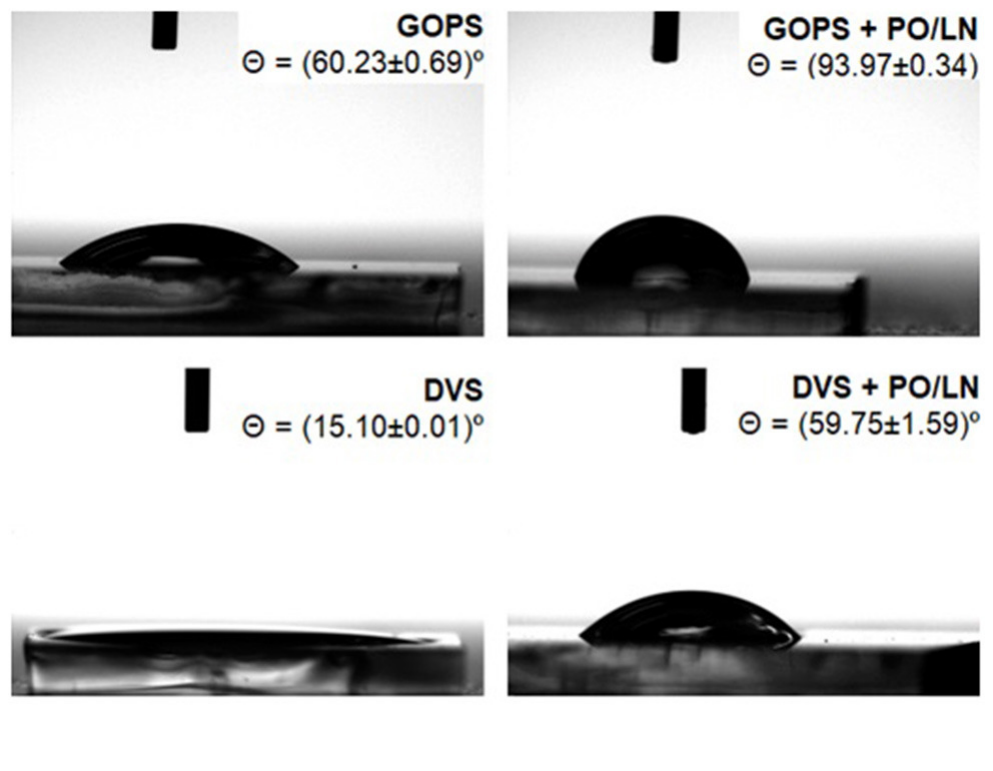
Contact angle images of water droplets deposited on PEDOT:DVS and PEDOT:GOPS samples before and after coating with Poly-ornithine/Laminin (PO/LN); *n* = 3 samples for each condition (each measurement was performed in triplicates).

Contact angle (Θ [°]) analysis allowed to evaluate the differences in surface wettability between GOPS- and DVS-cross-linked films, before and after PO/LN coating, being LN a ECM protein fundamental for NSCs adhesion. Wettability analysis allows us to establish whether a surface can be considered hydrophobic (Θ >90°) or hydrophilic (Θ <90°), which impacts initial cell adhesion and survival (Tamada and Ikada, 1993; Hornyak and Rao, 2016). PEDOT:DVS films obtained are more hydrophilic than PEDOT:GOPS ones, with contact angles of 15.10 ± 0.01° and 60.23 ± 0.69°, respectively (Figure 2). Moreover, surface coating with PO/LN leads to a decrease in hydrophilicity of both cross-linked films, but PEDOT:DVS film (59.75 ± 1.59°) continues to be more hydrophilic than PEDOT:GOPS (93.97 ± 0.34°).

### Stability of Cross-Linked PEDOT Films

**Figure 3:**
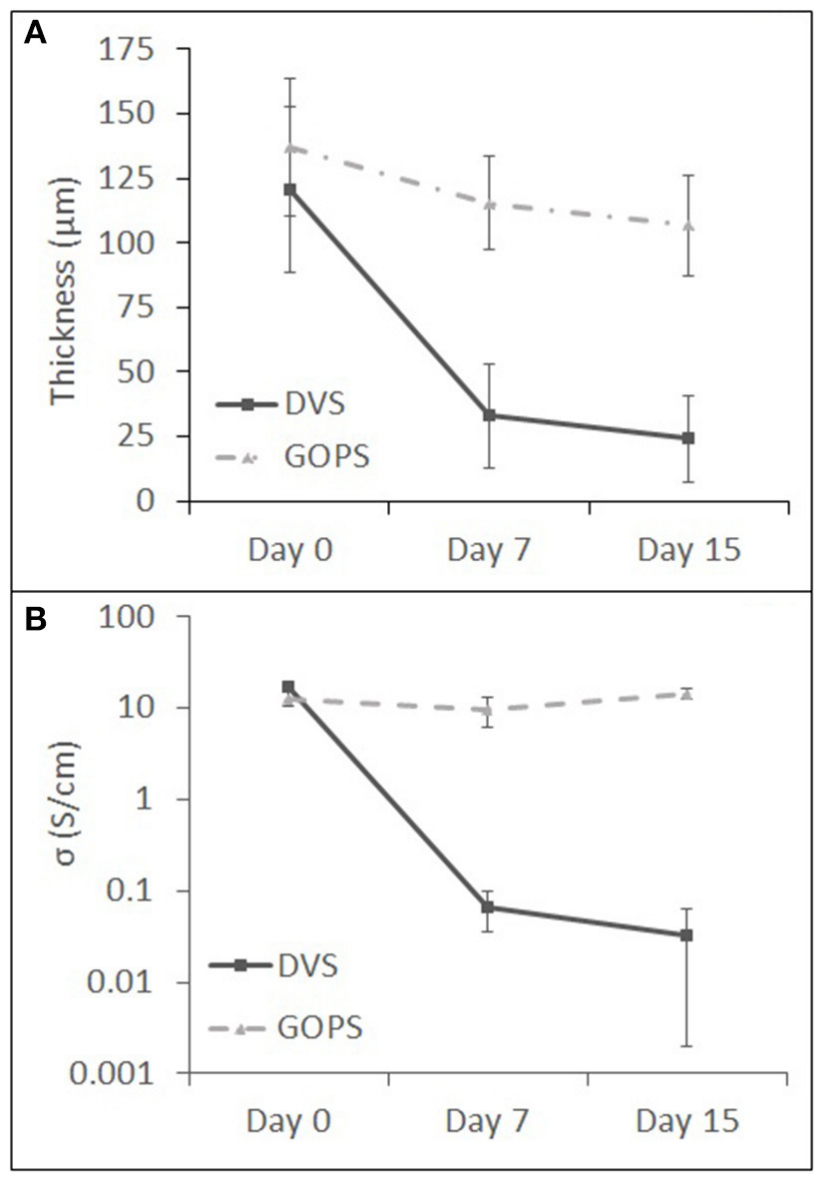
Variation in thickness **(A)** and conductivity **(B)** of PEDOT:GOPS and PEDOT:DVS films upon immersion in MilliQ water; *n* = 3 samples for each time point (each measurement was performed in triplicates).

Structural stability of cross-linked PEDOT:PSS films upon immersion in water was studied in this work. The goal was to assess possible changes in the film's properties. PEDOT:DVS and PEDOT:GOPS film samples were left immersed in MilliQ water for 7 and 15 days (Figure 3 and Table 2). Water immersion lead to changes in the thickness (Figure 3A) and electroconductivity (Figure 3B) of both films. PEDOT:DVS suffered an 80% thickness loss after 15 days, while PEDOT:GOPS shows only 22% loss. The conductivity followed a similar trend. A dramatic reduction of PEDOT:DVS conductivity occurs after 7 days (from an initial value of 17 to 0.07 S cm^−1^), which was then maintained after 15 days of immersion (0.03 S cm^−1^). The electroconductivity of PEDOT:GOPS films slightly decreases after 7 days (from 13 to 10 S cm^−1^) and increased again after 15 days (15 S cm^−1^). Despite the cross-linked nature of the PEDOT films, with the DVS-cross-linked ones evidencing a loser network, both films will probably swell upon prolonged contact with water or the culture medium electrolyte. In case of the loser network, material may by “re-suspended,” leading to a material loss of the film, with the concomitant thickness decrease. The loss in conductivity suggests that also doped PEDOT will be released from the film. In addition, the material may also delaminate from the supporting glass substrate. We do not expect that the flow of current through the PEDOT film will induce a degradation. The results obtained for the PEDOT:PSS:GOPS films support this conclusion.

**Table 2:** Thickness and conductivity values of PEDOT:PSS films cross-linked with GOPS and DVS measured after different immersion times in water (Day 0, Day 7, and Day 15) (mean ± std., *n* = 3).

	**Thickness (μm)**		**Conductivity σ (S cm^−1^)**	
	**DVS**	**GOPS**	**DVS**	**GOPS**
Day 0	121 ± 32	137 ± 27	17 ± 1	13 ± 2
Day 7	33 ± 20	115 ± 18	0.07	10 ± 3
Day 15	24 ± 16	107 ± 19	0.03	15 ± 2

Therefore, we can assume that PEDOT:GOPS films show a constant conductivity trend during time in water, with an approximate value of 12 S cm^−1^, contrary to PEDOT:DVS films. Moreover, the reduction in thickness in PEDOT:DVS samples suggests that material loss into the water occurs along the timeframe of our envisaged cell studies, contrary to what is observed for PEDOT:GOPS. In view of these results, PEDOT:GOPS was used as a substrate for the electrical stimulation of NSCs.

### Electrical Stimulation of ReNcell-VM—Metabolic Activity

**Figure 4:**
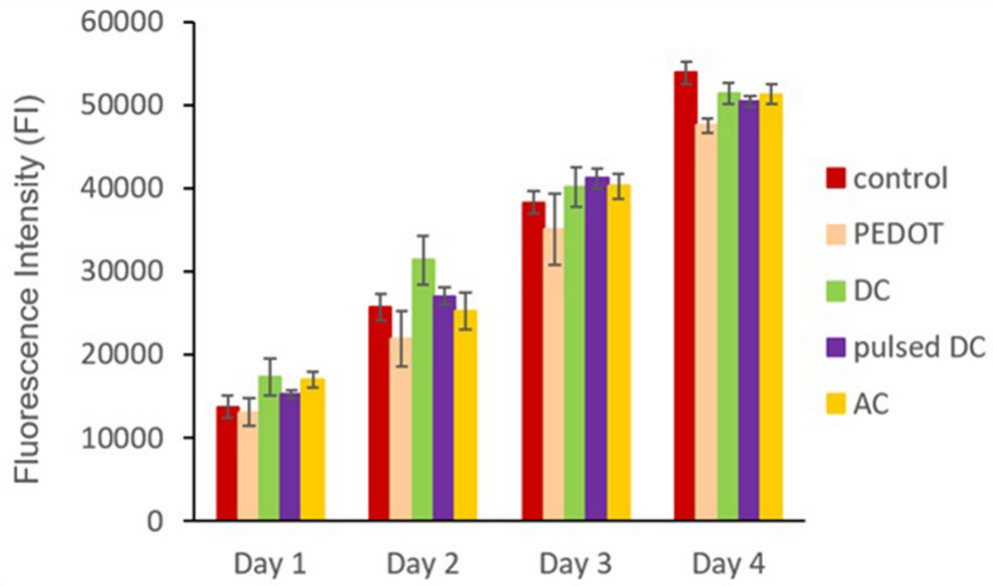
Fluorescence intensity of Alamar Blue® bioassay from ReNcells-VM cultured on PEDOT:GOPS films under different electrical stimulation conditions (DC, pulsed DC, and AC). “Control” corresponds to tissue culture plates and “PEDOT” to PEDOT:GOPS setups with no application of electrical stimulation; *n* = 2 replicates of the experiment (each experiment performed in triplicates for each tested condition).

After ReNcell-VM seeding onto the polymeric films, cell metabolic activity was assessed during the expansion phase (24 h stimulation per day, N2 expansion medium) by Alamar Blue® viability assay (Figure 4). ReNcell-VM cells on PEDOT:GOPS were exposed to different electrical stimulation conditions (DC, pulsed DC, and AC). Standard tissue culture plates and PEDOT:GOPS setups without electrical stimulation were used as controls.

Results show that the different electrical stimulation protocols used do not compromise ReNcells-VM metabolic activity throughout the time period assessed. All samples show a constant increase in fluorescence intensity, suggesting cell number increase. All samples with applied electrical stimulation show slightly higher values, but without statistically significant differences from the samples without electrical stimulation.

### Electrical Stimulation of ReNcell-VM—Differentiation

The effect of electrical stimulation on ReNcell-VM differentiation (8 days) profile was evaluated using immunofluorescence and qRT-PCR. The ReNcells-VM differentiation into neuronal and glial lineages was promoted by growth factor removal and switching to N2B27 medium (Donato et al., 2007).

**Figure 5:**
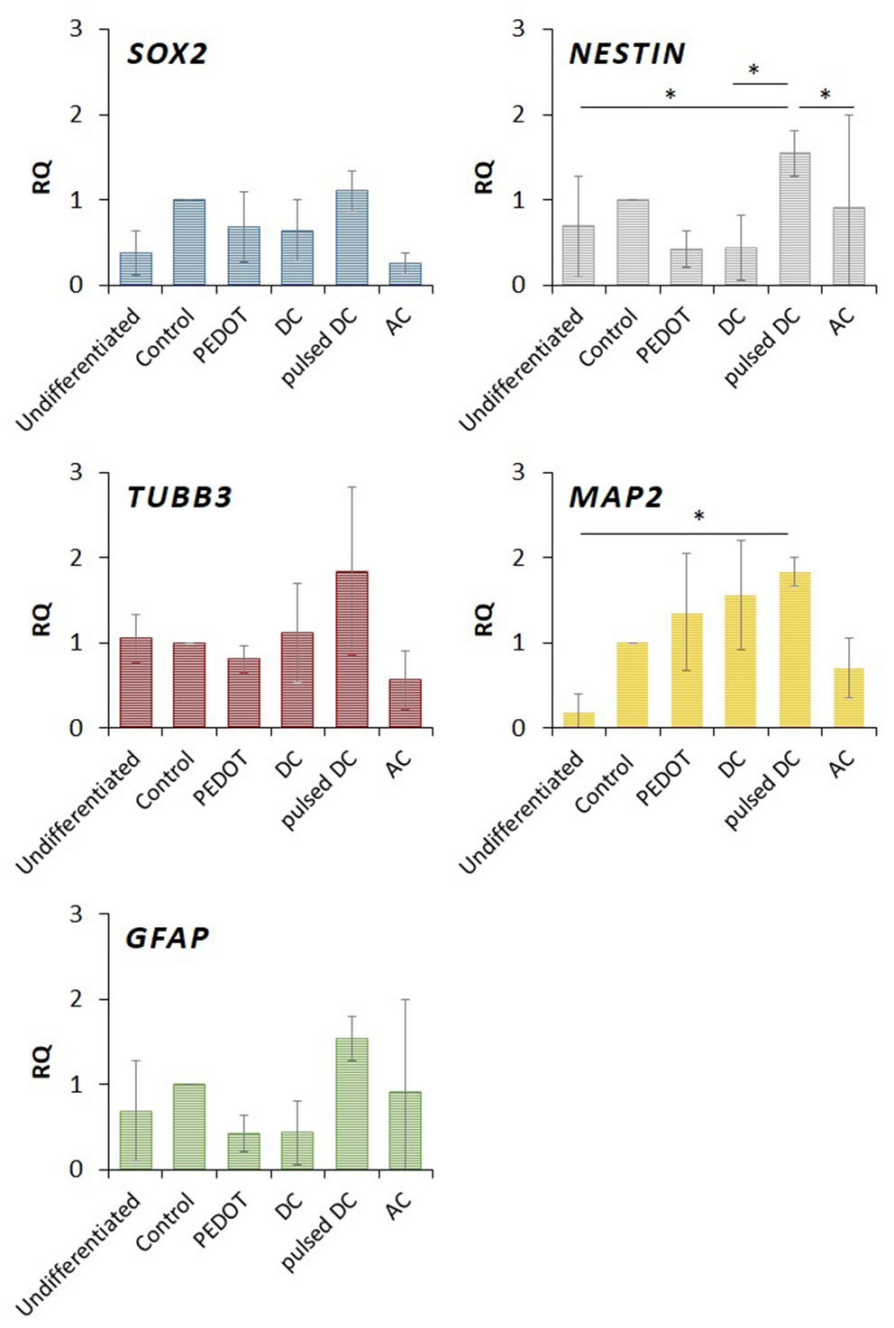
Profiles of gene expression obtained with qRT-PCR on ReNcells-VM after 8 days of differentiation under various electrical stimulation conditions (DC, pulsed DC, and AC). The following genes were considered: SOX2 and NESTIN (stem/progenitor cells), TUBB3 (early neurons), MAP2 (mature neurons), GFAP (glial cells) (mean ± std., *n* = 2) replicates of the experiment (each experiment performed in triplicates for each tested condition), *p-value < 0.05.

qRT-PCR was performed to analyze gene transcription activity at the end of the differentiation protocol under the various electrical stimulation patterns. The results of qRT-PCR for SOX2, NESTIN, TUBB3 (the gene encoding for TUJ1), GFAP, and MAP2 are depicted in Figure 5. It also includes the cells collected at the end of the expansion phase, and differentiated on the tissue culture plate and on PEDOT:GOPS setups without electrical stimulation. The expression of SOX2 is similar for all conditions, but slightly decreased for AC electrical stimulation. Considering NESTIN, pulsed DC stimulation shows a higher expression compared to the other electrical stimulation conditions and to undifferentiated ReNcells-VM, with statistical significance (*p* < 0.05). These results indicate that ReNcells-VM still exhibit expression of neural progenitor genes after 8 days of differentiation.

Cells stimulated with pulsed DC showed increased expression of the differentiation genes tested (TUBB3, MAP2, and GFAP) in relation to the other conditions. The expression of mature neurons (MAP2), under pulsed DC, was significantly increased with respect to undifferentiated ReNcells-VM (*p* < 0.05). MAP2 also increased on DC stimulation and PEDOT:GOPS films without electrical stimulation. TUBB3 expression was enhanced by DC and pulsed DC stimulation.

**Figure 6:**
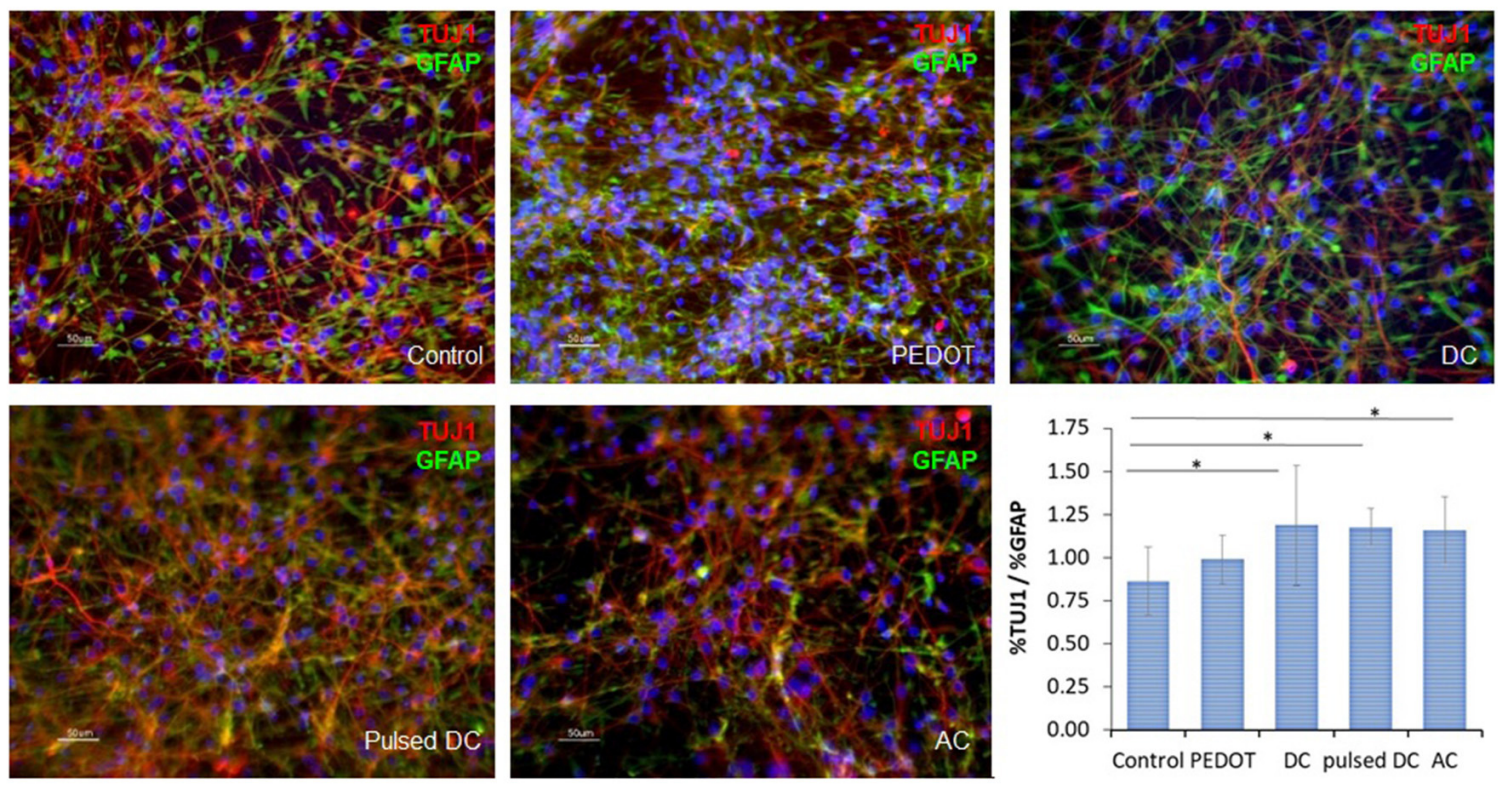
Immunofluorescence of ReNcells-VM cultured under different electrical stimulation conditions (DC, pulsed DC, and AC), after 4 days of expansion and 8 days of differentiation, stained for TUJ1 (red), GFAP (green), DAPI (blue). Cultures on standard tissue culture plates (“Control”) and on PEDOT:GOPS without electrical stimulation (“PEDOT”) were used as controls. Ratio between TUJ1 % and GFAP % calculated using the total image area (bottom right panel); *n* = 8 ratios for each condition, *p-value < 0.05, scale bar: 50 μm.

Cells were immunostained for the markers TUJ1 and GFAP to evaluate the differentiation into specific neural phenotypes. Results for immunostaining are shown in Figure 6. All cultures show positive staining for both markers. The percentage of TUJ1 and GFAP was quantified using ImageJ and the ratio TUJ1/GFAP was calculated to compare neuronal against glial differentiation under the different electrical stimulation conditions. The results (Figure 6) show higher ratios for cultures under electric stimulation (DC, pulsed DC, or AC stimulation), with statistical significance (*p* < 0.05). TUJ1/GFAP ratio is higher than 1 for all electrical stimulation conditions, suggest that a slightly higher number of neurons is present in culture than astrocytes. For PEDOT:GOPS films without electrical stimulation the ratio is equal to 1, meaning equal quantities of neurons and astrocytes coexist in the non-stimulated setup, and for the tissue culture plate the ratio is <1, suggesting slightly higher number of astrocytes in the culture.

## Discussion

### Preparation of Cross-Linked PEDOT:PSS Films

The preparation of electroconductive substrates is critical for neural tissue engineering applications using electrical stimulation (Luo et al., 2008; Balint et al., 2014; Guo and Ma, 2018). However, limited comparison exists between the electrical stimulation protocols available. For such study, a reliable electroconductive platform is required. PEDOT:PSS was our choice as it is one of the most stable, safe and versatile electroconductive polymers available (Luo et al., 2008; Shi et al., 2015; Heo et al., 2019). Commercially available PEDOT:PSS aqueous dispersions can be used as received to prepare PEDOT:PSS films with a wide range of electroconductivities. However, without further treatment, such films will re-suspend in water, which limits their use as scaffolds on aqueous environments, namely to support neural cell cultures. Such limitation can be addressed by the use of appropriate cross-linking protocols able to stabilize PEDOT:PSS films in aqueous environments, while retaining or improving their electroconductive and biocompatible properties (Shi et al., 2015). GOPS has been reported as a cross-linking agent able to yield a biocompatible and stable PEDOT:PSS film, but contributing also to reduce its conductivity (Balint et al., 2014; Håkansson et al., 2017). In this work, the performance of two different cross-linking protocols, based on distinct cross-linking agents (GOPS and DVS) was compared. Two different starting dispersions were prepared with the same amount of PEDOT:PSS (as received dispersion) and EG (1:4 by volume), adding a percentage of cross-linker and secondary dopant (DBSA) as described in literature, namely 1% (v/v) of GOPS and 0.05% (v/v) of DBSA for the first solution (Pires et al., 2015) and 3% (v/v) of DVS and 0.1% (v/v) of DBSA for the second one (Mantione et al., 2017). Annealing process was carried on following the same protocols described in literature (Pires et al., 2015; Mantione et al., 2017).

DVS was used as a cross-linking agent for PEDOT:PSS films, which can also act as a secondary dopant, to increase films electroconductivity (Mantione et al., 2017). The performance of GOPS and DVS cross-linked PEDOT:PSS was compared by Mantione et al. using a protocol slightly different from ours. In this previous study it was used a different PEDOT:PSS formulation, with lower PSS content and higher conductivity. Moreover, in such study, for the preparation of cross-linked PEDOT:PSS with GOPS, a 1:4 volume ratio of EG to PEDOT:PSS was also used, but a higher DBSA concentration (1.0 vs. 0.5 μL mL^−1^), and various percentages of GOPS (from 1 to 5%) were studied. GOPS cross-linked PEDOT:PSS films were prepared by Mantione et al. at 1,000 rpm for 40 s and annealed at 140°C for 40 min for conductivity measurements, while for cell culture the films were annealed at 50°C for 60 min under vacuum. For the corresponding PEDOT:DVS samples, 1:16 volume parts of EG to PEDOT:PSS were used but with the same DBSA content as used in this study (1.0 μL mL^−1^) and different percentages of DVS, from 0 to 8%, were studied using conductivity measurements. In the end, films were prepared at 1,000 rpm for 40 s and annealed at 140°C for 40 min for conductivity measurements, while for cell culture films were annealed at 50°C for 60 min under vacuum. In our study we used the same annealing conditions for the samples used in the conductivity studies and in the cell culture. Furthermore, the aqueous stability of PEDOT:GOPS and PEDOT:DVS films was not reported by Mantione et al. Therefore, we decided in our study to compare and further characterize the cross-linked films obtained with respect to the critical properties for NSC cell culture: hydrophilicity, electroconductivity, and stability in water.

### Characterization of Cross-Linked PEDOT:PSS Films

The surface properties of a biomaterial are responsible for its interplay with cells, fluids, and components of the extracellular matrix (e.g., adhesion proteins; Menzies and Jones, 2010). Thus, biocompatibility can be highly influenced by material surface properties. In this study, PEDOT:GOPS films are more hydrophilic than PEDOT:DVS samples (Figure 2), being the contact angles reduced upon coating of the surfaces with PO/LN. We can, therefore, assume that the nature of the cross-linker used is the main determinant of the material surface properties. The contact angle values we obtained were previously described to be favorable to the adhesion of NSCs (Arima and Iwata, 2007; Tian et al., 2016), and therefore, considering also the stability upon contact with the culture medium, we pursued our studies with the PEDOT:GOPS substrates.

Electroconductive conjugated polymers such as PEDOT possess delocalized π orbitals that enable electrons mobility. Therefore, the more electroconductive materials enable a more efficient electrical stimulation of the cells. No threshold electroconductivity value exists in the literature for the application of conductive materials in cells electrical stimulation, but it is generally assumed that its value has to at least exceed that of the culture medium (0.01 S cm^−1^) (Mazzoleni et al., 1986). We obtained high electroconductivity values (Table 2) for our PEDOT:GOPS (13 ± 2 S cm^−1^) and PEDOT:DVS (17 ± 1 S cm^−1^) samples, all of them above 0.01 S cm^−1^.

The electroconductivity of the PEDOT:GOPS sample is higher than the value reported by Pires et al. (2015) for their films, and we hypothesize this is related to variations in the composition of the PEDOT:PSS water dispersion and/or details of the samples preparation. As for PEDOT:DVS, the electroconductivity obtained (17 ± 1 S cm^−1^) is lower than that previously obtained by Mantione et al. (2017) (~600 S cm^−1^). This is explained by the use of different PEDOT:PSS dispersions: we used Clevios P VP Al 4083, with a PEDOT:PSS ratio of 1:6, while Mantione et al. used Clevios PH 1000, with a PEDOT:PSS ratio of 1:2.5, a more conductive formulation (Mantione et al., 2017).

The stability of PEDOT:GOPS and PEDOT:DVS films in water was determined to evaluate their suitability for cell culture applications. Our results indicate that, for the materials preparation conditions employed, GOPS maintained film integrity in wet environments for long incubation periods (15 days) in contrast to DVS. This is evidenced by the maintenance of film's electrical properties and average thickness. Supporting evidence of these findings has been reported (ElMahmoudy et al., 2017), demonstrating that a concentration of 1% wt of GOPS can promote high electrical conductivity, sufficient mechanical stability, and steady performance over 3 weeks.

We found that PEDOT:GOPS and PEDOT:DVS films have different stability profiles in water (Table 2). GOPS is a bifunctional organosilane with three methoxy groups on one side and an epoxy ring on the other, whereas DVS is a sulfone compound with two S-vinyl substituents. The differences observed might be explained by the different cross-linking mechanisms involved for GOPS and DVS, a matter that is still under debate. The cross-linking mechanism with PEDOT has been reported to be similar for both GOPS (Håkansson et al., 2017) and DVS (Jennings et al., 2018), where the epoxy and vinyl groups, respectively, react with the sulfonate group of PSS. This process allows the establishment of bridges across different PSS molecules, forming a network that is responsible to stabilize the obtained PEDOT film. The main difference between them is that GOPS can also establish Si-O-Si bonds with other GOPS molecules extending the network density, or even with the supporting glass to further anchor the film and reduce delamination (Håkansson et al., 2017; Solazzo et al., 2019), whereas DVS cannot (Mantione et al., 2017; Bora et al., 2019). In addition, the ethylene glycol used in the two cross-linkable formulations, may also undergo a condensation reaction with Si-OCH_3_ group, further extending the network. Our results show that, under the conditions we have used, the network established in PEDOT:GOPS films is chemically more stable and resistant to erosion and delamination when compared to PEDOT:DVS, leading to an improved stability of PEDOT:GOPS in water. As such, PEDOT:GOPS films were used for our studies on electrical stimulation of NSCs.

### Electrical Stimulation of ReNcell-VM

NSCs in their cell niche undergo asymmetric division generating two daughter cells, one that is identical to the original cell and one that is programmed to differentiate into a non-stem cell. When this mechanism is induced by external cues provided to the precursor cell, it is called extrinsic asymmetric cell division. Depending on their position in the stem cell niche, the daughter cells may acquire different fates owing to exposure to varying external signals (Morrison and Kimble, 2006). We hypothesize that the combination of chemical and pulsed electrical cues is able to better mimic the complexity of the neural stem cell niche *in vivo* and that plays a key role in the differentiation mechanism of NSCs.

PEDOT:GOPS films were used to study the effect of different electrical stimulation protocols on ReNcell-VM metabolic activity and differentiation. Results from Alamar Blue® Assay (Figure 4) demonstrate that all samples show a constant increase in fluorescence intensity, suggesting cell number increase. No differences were found regarding cell growth during the expansion phase, indicating the absence of toxic effects on the cells upon use of both PEDOT:GOPS platform and the electrical stimulation protocols. The positive effect of the electrical stimulation on NSCs growth and proliferation has been widely described in the literature (Yamada et al., 2007; Chang et al., 2011; Huang et al., 2015; Zhu et al., 2019). However, our results suggest that none of the protocols tested produced significant effects on ReNcell-VM metabolic activity during the first 4 days of stimulation.

At the end of the differentiation phase, qPCR and Immunofluorescence analysis were performed. qPCR analysis revealed a trend in gene expression: electrical stimulation by DC and pulsed DC was associated with higher expression of NESTIN, TUBB3, and MAP2. Among the electrical stimulation protocols tested, statistically significant increases in gene expression were found for NESTIN and MAP2 expression in pulsed DC. This indicates that pulsed DC positively influences ReNcell-VM expression of neuronal differentiation marker genes. Differences in TUBB3 and GFAP expression were found for the different various electrical stimulation protocols tested, but these were not statistically significant.

Immunofluorescence analysis was performed to evaluate ReNcell-VM morphology and neuron/astrocyte marker expression. In this study, we focused our analysis on the specific cell marker analysis for neurons (Tuj1) and astrocytes (GFAP). The images obtained (Figure 6A) show elongated cells with numerous projections, indicating that the differentiation of ReNcell-VM was successful. The relative quantification of the proteins TUJ11 and GFAP was then performed by immunostaining to investigate the effect of electrical stimulation on cell fate. The higher number of TUJ1 positive over GFAP positive staining in electrical stimulation samples suggests a greater prevalence of neuronal- over glial-committed cells. These results show that the electrical stimulation has a positive effect on NSCs phenotypic expression of markers of differentiation toward neuronal lineage. A similar trend has also been shown for MAP2 (neurons) and GFAP (astrocytes) expression cells when differentiated on flat titanium substrates under electrical stimulation (Yang et al., 2017). Other studies in the literature report similar trends (Chang et al., 2011; Kobelt et al., 2014; Pires et al., 2015; Stewart et al., 2015).

Immunofluorescence results were in accordance with the results of qRT-PCR. The analysis focused on the relative expression of SOX2 and NESTIN (neural stem/progenitor cells), and three genes that are expressed during cell differentiation: TUBB3 (early neurons), MAP2 (intermediate neurons), and GFAP (glial cells). Specifically, the transcription factor SOX2 is expressed in neural progenitor populations in the developing central nervous system and it is necessary to maintain their progenitor identity (Hutton and Pevny, 2011). NESTIN is an intermediate filament protein that is known as a neural stem/progenitor cell marker, expressed in undifferentiated central nervous system cells during development (Suzuki et al., 2010). During neuro- and glio-genesis, NESTIN is replaced by cell type-specific intermediate filaments, such as neurofilaments and GFAP. GFAP is the main intermediate filament protein in mature astrocytes, but also an important component of the cytoskeleton in astrocytes and immature neurons during development (Casper and McCarthy, 2006; Dráberová et al., 2008; Middeldorp and Hol, 2011). Finally, the expression of the genes TUBB3 and MAP2 are typical neuron-specific markers. TUJ1 (encoded by TUBB3 gene) is the protein that provides stability to microtubules in neuronal cell bodies and axons, and it is present in the early stages of neuron development (Huang et al., 2012). MAP2 belongs to a family of proteins responsible for stabilizing neuronal shape by promoting microtubule synthesis (Morrison et al., 1998). MAP2 expression is only observed in the middle to late stages of neuron development.

To summarize, ReNcells-VM are capable of differentiating into a higher number of neurons in a non-stimulated PEDOT:GOPS substrate then on standard tissue plate culture plates, but this effect is further enhanced when cultures on PEDOT:GOPS are electrically stimulated. DC and pulsed DC stimulation have a similar impact on ReNcell-VM differentiation toward neurons, as demonstrated by immunofluorescence. Moreover, AC stimulation failed to show significant impact in the expression of both neuronal and astrocytic markers. Pulsed DC electrical stimulation, as demonstrated also by immunofluorescence analysis, was the condition that led to higher efficiency of ReNcell-VM differentiation toward neuronal lineage. We hypothesize that this type of stimulus better suits the conditions for higher production of neural cells for tissue engineering applications, being even similar to the conditions observed *in vivo* (Mazaheri and Jensen, 2010).

## Conclusion

In this study PEDOT:GOPS and PEDOT:DVS films were compared. PEDOT:GOPS films were found to have higher contact angle (60.23 ± 0.69° vs. 15.1 ± 0.01°), similar electroconductivity (13 ± 2 S cm^−1^ vs. 17 ± 1 S cm^−1^) and increased stability for 15 days in water when compared to PEDOT:DVS. PEDOT:GOPS was used as a platform to study the effect of different electrical stimulation protocols (AC, DC, and pulsed DC) on ReNcell-VM metabolic activity and differentiation (1 V cm^−1^). qRT-PCR results suggest that pulsed DC stimulation enhances NSCs neuronal differentiation. The ratio of TUJ1/GFAP markers expression was significantly higher for all electrical stimulation conditions. With this study, we demonstrate that differences in electrical cues affect NSCs fate. PEDOT:PSS cross-linked substrates coupled with pulsed DC electrical stimulation are powerful candidates for mimicking stem cell niches for tissue engineering applications. We believe that these findings also have relevant implications for PEDOT-PSS coating of deep brain electrodes for application in neurodegenerative disorders.

## Data Availability Statement

The raw data supporting the conclusions of this article will be made available by the authors, without undue reservation.

## Author Contributions

LS, CR, FF, and JM: original idea. LS, FG, CR, FF, and JM: experimental plan. LS assisted by FF and JM: experimental work on materials. LS assisted by CR and FF: experimental work with cell culture. LS assisted by FG and CR: cell characterization. LS and FG: qRT-PCR. LS with guidance by CR, FG, JM, and FF: data analysis and figures preparation. CR, RL, JC, FF, and JM: scientific guidance and discussions, laboratory space, and funding. LS and FG: manuscript writing and original drafting. CR, RL, JC, JM, and FF: writing—review and editing. All authors revised the manuscript.

## Conflict of Interest

The authors declare that the research was conducted in the absence of any commercial or financial relationships that could be construed as a potential conflict of interest.
