# Supplementary material for: Effect of Electrical Stimulation Conditions on Neural Stem Cells Differentiation on Cross-Linked PEDOT:PSS Films
Source: Front Bioeng Biotechnol. 2021 Feb 12;9:591838. doi: 10.3389/fbioe.2021.591838 (PMC7928331; doi:10.3389/fbioe.2021.591838)
Supplement: Supplementary file 1 [file Data_Sheet_1.docx]

**SUPPLEMENTARY INFORMATION**

1. **SEM pictures of cross-linked PEDOT:PSS films**


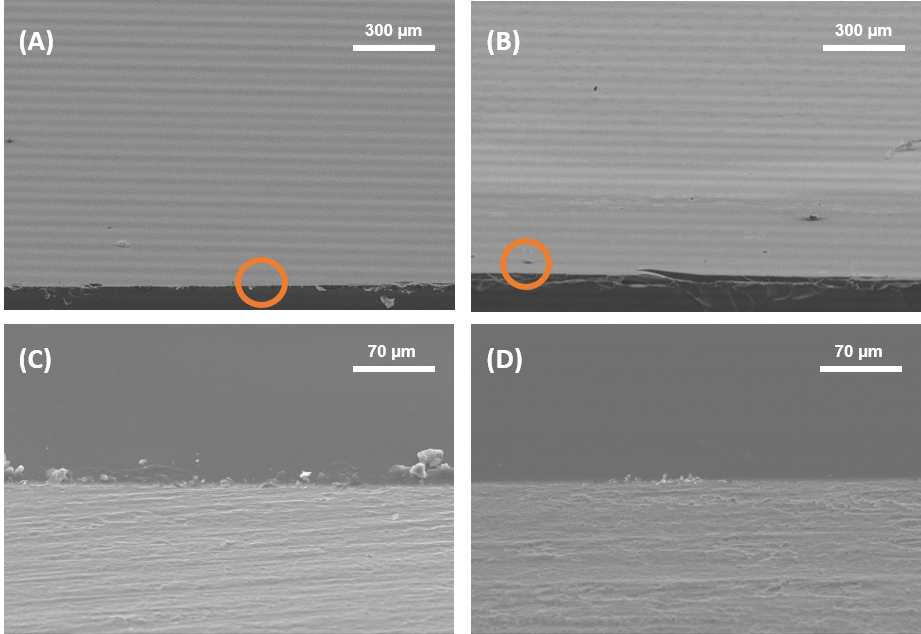


**Fig. S1**: SEM pictures of PEDOT:PSS films cross-linked with GOPS (A) and DVS (B) and respective zoom (area in the orange circle) of the transversal section for GOPS cross-linked (C) and DVS-crosslinked (D) films.

1. **FTIR spectra**


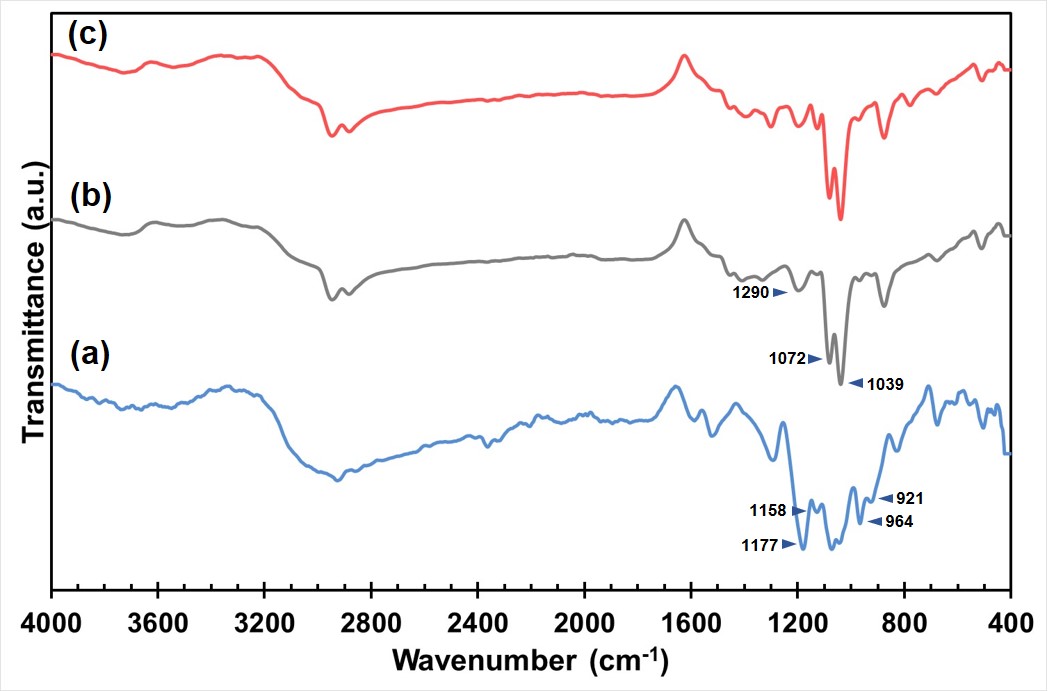


**Fig. S2**: FTIR transmittance spectra (resolution of 4 cm^-1^, 32 scans) of (a) the original PEDOT solution and after mixture with the cross-linkers (b) GOPS and (c) DVS.

1. **ReNcells-VM morphology before and after differentiation**


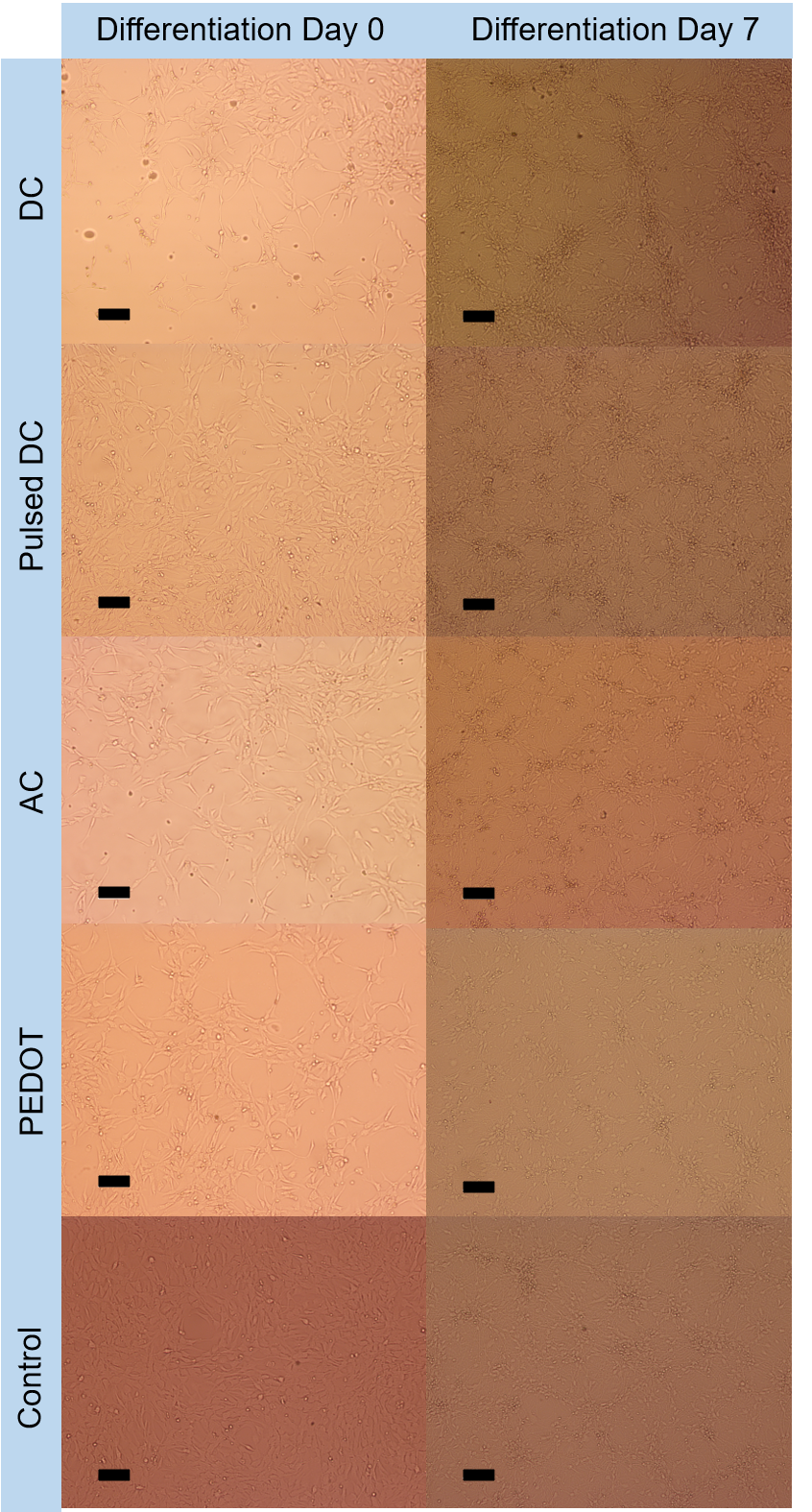


**Fig. S3**: ReNcells-VM morphology at Day 0 and Day 7 of the differentiation phase for all tested conditions (DC, pulsed DC and AC electrical stimulation, PEDOT films with no applied stimulation, tissue culture plates (control)). Optical microscope, scale bar: 100 µm.

1. **Immunofluorescence analysis**

Immunofluorescence quantitative analysis was performed by immunostaining every sample of every condition for TUJ1 and GFAP and counterstained for DAPI. Number of cells was quantified by nuclei staining for DAPI. Several pictures were acquired, with both biomarkers in order to obtain the merged images presented in Figures 6. The analysis was then performed through the software ImageJ. Every picture was made binary by the software and the total area of the picture stained for TUJ1 or GFAP was quantified. This way, when showing the expression levels of TUJ1 and GFAP, those were estimated for the same number of cells present in that picture. The TUJ1/GFAP ratio was calculated by EQ.1:

$\frac{TUJ1}{GFAP} ratio=\frac{\frac{\% area TUJ1}{n. of cells}}{\frac{\% area GFAP}{n. of cells}}=\frac{\% area TUJ1}{\% area GFAP}$ EQ.S1

The calculated ratios for every condition are presented in Table S1.

Table S1: Calculated number of cells and percentage of area stained for immunofluorescence markers for neurons (TUJ1) and astrocytes (GFAP), for every tested condition (DC, pulsed DC, AC, PEDOT, Control) and respective ratio %TUJ1/%GFAP.

| Sample | N. OF CELLS | % area TUJ1 | % area GFAP | RATIO |
| --- | --- | --- | --- | --- |
| DC | 358 | 35.67 | 37.53 | 0.95 |
| DC | 668 | 51.21 | 41.45 | 1.24 |
| DC | 241 | 42.43 | 40.60 | 1.05 |
| DC | 159 | 20.69 | 19.03 | 1.09 |
| DC | 170 | 34.91 | 33.46 | 1.04 |
| DC | 442 | 24.44 | 42.59 | 0.57 |
| DC | 926 | 68.62 | 43.87 | 1.56 |
| DC | 296 | 54.11 | 31.81 | 1.70 |
| DC | 318 | 48.19 | 32.48 | 1.48 |
|  |  |  |  |  |
| Pulsed DC | 2874 | 74.55 | 58.90 | 1.27 |
| Pulsed DC | 641 | 57.08 | 50.34 | 1.13 |
| Pulsed DC | 849 | 60.45 | 44.92 | 1.35 |
| Pulsed DC | 918 | 44.61 | 42.23 | 1.06 |
| Pulsed DC | 1111 | 32.94 | 27.07 | 1.22 |
| Pulsed DC | 901 | 46.31 | 44.37 | 1.04 |
| Pulsed DC | 813 | 49.13 | 42.10 | 1.17 |
|  |  |  |  |  |
| AC | 356 | 55.89 | 58.58 | 0.95 |
| AC | 93 | 51.38 | 34.24 | 1.50 |
| AC | 835 | 42.16 | 39.97 | 1.05 |
| AC | 1114 | 61.5 | 54.88 | 1.12 |
| AC | 221 | 51.85 | 50.59 | 1.02 |
| AC | 302 | 56.19 | 40.62 | 1.38 |
| AC | 422 | 47.60 | 47.01 | 1.01 |
| AC | 602 | 51.41 | 41.92 | 1.22 |
|  |  |  |  |  |
| PEDOT | 135 | 6.55 | 7.92 | 0.83 |
| PEDOT | 565 | 9.46 | 10.19 | 0.93 |
| PEDOT | 579 | 32.86 | 33.49 | 0.98 |
| PEDOT | 101 | 16.34 | 18.22 | 0.90 |
| PEDOT | 139 | 19.02 | 22.00 | 0.86 |
| PEDOT | 773 | 45.92 | 37.66 | 1.22 |
| PEDOT | 120 | 20.02 | 19.29 | 1.04 |
| PEDOT | 148 | 26.47 | 22.69 | 1.17 |
|  |  |  |  |  |
| Control | 1441 | 37.72 | 56.75 | 0.66 |
| Control | 1272 | 37.73 | 60.79 | 0.62 |
| Control | 1323 | 37.39 | 57.29 | 0.65 |
| Control | 1345 | 41.62 | 54.79 | 0.76 |
| Control | 382 | 40.80 | 54.13 | 0.75 |
| Control | 439 | 40.57 | 52.35 | 0.77 |
| Control | 712 | 60.54 | 48.58 | 1.25 |
| Control | 194 | 46.61 | 47.49 | 0.98 |
| Control | 233 | 38.43 | 42.73 | 0.90 |
| Control | 269 | 52.63 | 45.28 | 1.16 |
| Control | 314 | 51.47 | 46.34 | 1.11 |
| Control | 1301 | 51.29 | 57.37 | 0.89 |
| Control | 395 | 37.82 | 49.79 | 0.76 |
| Control | 499 | 42.53 | 51.61 | 0.82 |

The several ratios obtained from every sample were averaged in order to quantify if the expression of TUJ1 (neuronal lineage) was higher (ratio > 1), lower (ratio < 1) or equal (ratio = 1) to that of GFAP (glial lineage). Averaged results are shown in Table S2. Using this approach, we hope to make it easy for the reader to understand which specific condition of electrical stimulation gave a higher number of neurons in relation to glial cells. Moreover, with this method, we avoided any mistake due to the picture (for example, imaging just a part of a cell) and we managed to make calculations even with a high number of cells for picture.

Table S2: Averaged ratios calculated from every immunofluorescence picture for every tested condition, in order to quantify if the expression of TUJ1 (neuronal lineage) was higher (ratio > 1), lower (ratio < 1) or equal (ratio = 1) to that of GFAP (glial lineage).

| Sample | Average | STD |
| --- | --- | --- |
| DC | 1.19 | 0.35 |
| Pulsed DC | 1.18 | 0.11 |
| AC | 1.16 | 0.19 |
| PEDOT | 0.99 | 0.14 |
| Control | 0.86 | 0.20 |
